# Determination of the Phenolic Profile by Liquid Chromatography, Evaluation of Antioxidant Activity and Toxicity of Moroccan *Erica multiflora, Erica scoparia*, and *Calluna vulgaris* (*Ericaceae*)

**DOI:** 10.3390/molecules27133979

**Published:** 2022-06-21

**Authors:** Douaa Bekkai, Yassine Oulad El Majdoub, Hamid Bekkai, Francesco Cacciola, Natalizia Miceli, Maria Fernanda Taviano, Emilia Cavò, Tomader Errabii, Roberto Laganà Vinci, Luigi Mondello, Mohammed L’Bachir El Kbiach

**Affiliations:** 1 Team of Plant Biotechnology, Biology Department, Abdelmalek Essaadi University, Tetouan 93000, Morocco; bekkai.douaa@gmail.com (D.B.); t.errabii@uae.ac.ma (T.E.); melkbiach@uae.ac.ma (M.L.E.K.); 2 Department of Chemical, Biological, Pharmaceutical and Environmental Sciences, University of Messina, 98166 Messina, Italy; youladelmajdoub@unime.it (Y.O.E.M.); nmiceli@unime.it (N.M.); mtaviano@unime.it (M.F.T.); ecavo@unime.it (E.C.); robertolaganavinci@gmail.com (R.L.V.); lmondello@unime.it (L.M.); 3 Chemistry Department, Abdelmalek Essaadi University, Tetouan 93000, Morocco; bekhamid1@hotmail.com; 4 Department of Biomedical, Dental, Morphological and Functional Imaging Sciences, University of Messina, 98125 Messina, Italy; 5 Chromaleont s.r.l., c/o Department of Chemical, Biological, Pharmaceutical and Environmental Sciences, University of Messina, 98166 Messina, Italy; 6 Department of Sciences and Technologies for Human and Environment, University Campus Bio-Medico of Rome, 00128 Rome, Italy

**Keywords:** *Ericaceae*, LC–DAD/ESI–MS, phenolic compounds, flavonoids, antioxidant activity, *Artemia salina* Leach

## Abstract

This study aimed to investigate the phenolic profile and selected biological activities of the leaf and aerial extracts of three *Ericaceae* species, namely *Erica multiflora*, *Erica scoparia*, and *Calluna vulgaris*, collected from three different places in the north of Morocco. The phenolic composition of all extracts was determined by LC coupled with photodiode array and mass spectrometry detection. Among the investigated extracts, that of *E. scoparia* aerial parts was the richest one, with a total amount of polyphenols of 9528.93 mg/kg. Up to 59 phenolic compounds were detected: 52 were positively identified and 49 quantified—11 in *C. vulgaris*, 14 in *E. multiflora*, and 24 in *E. scoparia*. In terms of chemical classes, nine were phenolic acids and 43 were flavonoids, and among them, the majority belonged to the class of flavonols. The antioxidant activity of all extracts was investigated by three different in vitro methods, namely DPPH, reducing power, and Fe^2+^ chelating assays; *E. scoparia* aerial part extract was the most active, with an IC_50_ of 0.142 ± 0.014 mg/mL (DPPH test) and 1.898 ± 0.056 ASE/mL (reducing power assay). Further, all extracts were non-toxic against *Artemia salina*, thus indicating their potential safety. The findings attained in this work for such Moroccan *Ericaceae* species, never investigated so far, bring novelty to the field and show them to be valuable sources of phenolic compounds with interesting primary antioxidant properties.

## 1. Introduction

*Ericaceae* is a cosmopolitan family, represented by 124 genera and 4100–4250 species that are widely distributed around the world, particularly in the Mediterranean area, in deficient and non-calcic soils, as well as in high mountains [1,2,3,4]. Within this family, *Erica* and *Calluna* are the most abundant and widely spread genera. In Northern Morocco, *E. multiflora*, *E. scoparia*, and *C. vulgaris* are traditionally consumed by local people in the form of infusions, and are well known for their therapeutic properties [5,6,7]. In Morocco, *Erica multiflora* L. and *Erica scoparia* L. are considered among the most well-known species of the *Erica* genus [1,8]. According to popular knowledge, both species might have anti-inflammatory and analgesic properties when it comes to urinary diseases [5,6]. Moreover, *E. multiflora* has shown antihyperlipidemic and liver function repair effects [8,9], and effective antilithiatic activity [10].

*Calluna vulgaris* (L.) Hull belongs to the monotypic genus of *Calluna*, also known for its powerful bioactive compounds. It is widely used to treat kidney and urinary system disorders, particularly inflammatory diseases of the bladder, prostate, and urinary tract [7,11,12,13,14,15]. It is also important to note that heather honey obtained from *C. vulgaris* nectar is a special type of honey that is highly appreciated by consumers, not only for its distinctive flavor and dietary value but also for its therapeutic purposes [12,15].

These biological effects are closely related to their composition in bioactive compounds such as flavonoids, tannins, anthocyanins, vitamins C and E, triterpenoids, saponins, proteins, steroids, coumarins, ascorbic acid, hydroquinone, etc. [4,16,17,18,19]. In the human body, the accumulation of free radicals induces numerous illnesses and health issues. Therefore, research within plants for natural antioxidant sources might be a promising alternative to lower the incidence of multiple diseases that are due to oxidative stress [20,21]. Polyphenols are an important class of secondary metabolites in plants, characterized by one or more hydroxyl groups binding to one or more aromatic rings, and are divided into two groups: flavonoids and non-flavonoids [22]. The biological and medicinal proprieties of antioxidant compounds such as plant polyphenols have been widely reported in the scientific literature [23]. Indeed, the protective role of polyphenols, especially as free radical scavengers, has been well established, and these molecules may play a prominent role in the prevention and/or the treatment of oxidative stress-induced diseases [24].

In the current study, *E. multiflora*, *E. scoparia*, and *C. vulgaris*, collected from Northern Morocco, were investigated for their phenolic composition and were further tested for their antioxidant properties as well as for their potential toxicity. In particular, the qualitative–quantitative profile of the phenolic constituents contained in the hydroalcoholic extracts obtained from the leaves and aerial parts of both *Erica* species and from the leaves of *C. vulgaris* was determined by LC–DAD/ESI–MS analyses. In order to provide a comprehensive view of the antioxidant profiles, the in vitro antioxidant effectiveness of the extracts was assessed by using three different methods: the DPPH (1,1-diphenyl-1-picrylhydrazyl) test and the reducing power and ferrous ion chelating assays. Moreover, the brine shrimp (*Artemia salina* Leach) lethality bioassay was utilized to evaluate the toxicity.

The phenolic content of *E. multiflora* has been already evaluated in other works [2,8,25,26]; however, either the leaves [2,8] or flowers [25]/entire plant [26] have been investigated. Notably, ref. [2] refers to an Algerian species, whereas ref. [25] refers to a Tunisian species. No data are available in the literature on the chemical composition and biological properties of *E. scoparia*; on the other hand, for *C. vulgaris*, only the inflorescences of a Portuguese species [27] have been reported so far.

## 2. Results and Discussion

### 2.1. Polyphenol Composition

**Figure 1:**
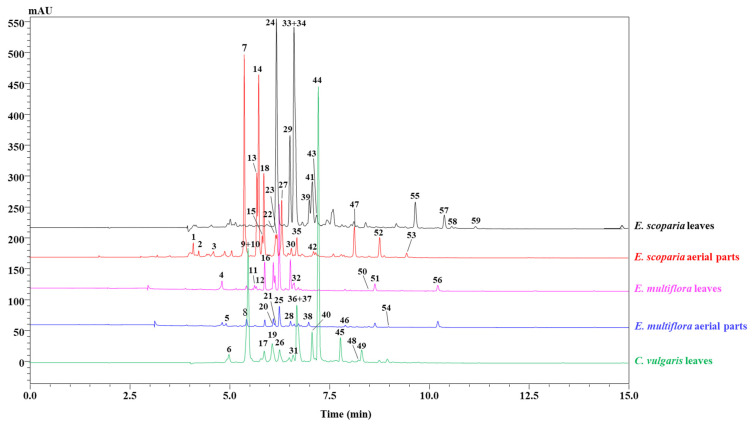
Chromatographic profile of hydroalcoholic extracts from leaves and aerial parts of 3 different *Ericaceae* taxa at λ = 330 nm.

The phenolic compounds present in the aerial parts and leaves of *C. vulgaris*, *E. multiflora*, and *E. scoparia* were identified by using an HPLC chromatogram at 330 nm (Figure 1). The main phenolic compounds were recognized by combining the retention times, UV spectra, and mass spectra of each peak with its standard, when available, and with literature data. The results revealed different quali-quantitative profiles among the studied parts, as shown in Figure 1. A total of 59 phenolic compounds were detected: 14 in *C. vulgaris*, 18 in *E. multiflora*, and 27 in *E. scoparia* (Table 1). Among them, 52 were positively identified (11 in *C. vulgaris*, 14 in *E. multiflora*, and 24 in *E. scoparia*). In terms of chemical classes, nine were phenolic acids and 43 were flavonoids, and among them, the majority belonged to the class of flavonols, mainly derivates of quercetin, myricetin, isorhamnetin, and kaempferol, while the rest of the compounds belonged to the class of flavanones, specifically eriodictyol and taxifolin. It is worth mentioning that, to the best of our knowledge, no previous studies have investigated the chemical composition of *E. scoparia*.

*Calluna vulgaris* leaves contained a total amount of phenolic compounds of 1567.78 mg/kg, comprising caffeoylquinic acid, which was the most abundant phenolic compound (1180 ± 8.18 mg/kg), followed by myricetin-*O*-rhamnoside (232.98 ± 0.30 mg/kg), myricetin-*O*-pentoside (48.81 ± 2.22 mg/Kg), and myricetin-*O*-hexoside (41.66 ± 1.88 mg/kg), whereas quercetin-*O*-hexoside (2.82 ± 3.24 mg/kg) was the lowest one. The results are in accordance with those presented by Mandim et al. [27] at the qualitative level, except for catechin, isorhamnetin-3-*O*-glucoside, and isorhmnetin-*O*-rhamnoside, which were absent in this studied species. However, a notable difference has been shown at the quantitative level, which could be, at least in part, attributed to the different organ of the plant used in this study, viz. leaves instead of inflorescences.

The leaves of *E. multiflora* contained 399.01 mg/kg of phenolic compounds, and were characterized by the presence of a quercetin derivative, myricetin-*O*-hexoside, and quercetin-*O*-(6″-cinnamoyl)-hexoside, while the aerial parts contained 227.6 mg/kg of phenolic compounds, and were distinguished by the presence of 4-caffeoylquinic acid, methyl-ellagic acid hexoside, and eriodictyol-*O*-hexoside, wherein 4-caffeoylquinic acid was the main compound in the aerial parts, with 83.75 ± 0.74 mg/kg, and where kaempferol was the least prevalent compound, with 0.95 ± 1.84 mg/kg. According to these results, it can be concluded that *E. multiflora* leaves presented higher phenolic compound content when compared to the aerial parts. The output of heat map analysis showed that the leaves and aerial parts of *E. multiflora* were clustered together into the same group and displayed the following main compounds in common: quercetin-*O*-hexoside, kaempferol-rhamnosyl-hexoside, rutin, caffeoylquinic acid, and kaempferol-hexoside. Moreover, in both parts, the presence of small amounts of three other compounds, quercetin, dimethylquercetin, and kaempferol, was noted. These results contradict those obtained by Mandim et al. [27], where quercetin was the most abundant compound, followed by kaempferol. This discordance could be partially related to the time and the location of the harvest, and/or the extraction method. *Erica scoparia* aerial parts presented a total amount of polyphenols of 9528.93 mg/kg. The most abundant compounds identified were myricetin-*O*-hexoside (2130.25 ± 0.78 mg/kg), myricetin-*O*-rhamnoside (1625.89 ± 0.39 mg/kg), and myricetin-*O*-pentoside (852.85 ± 1.97 mg/kg), whereas quercetin-*O*-(6″-p-hydroxybenzoyl)-hexoside (91.34 ± 1.22 mg/kg) was the least abundant one. Notably, myricetin-*O*-hexoside was shown to be the greatest phenolic compound in the leaves of *E. scoparia* (184.38 ± 0.26 mg/kg), while the smallest content was recorded for quercetin-*O*-(malonyl)-hexoside (18.52 ± 0.27 mg/kg). Thus, a remarkable discrepancy in the phenolic composition between the leaves and aerial parts of *E. scoparia* was observed. In addition, some phenolic compounds contained in the aerial parts seemed to be entirely absent in the leaves, such as taxifolin, digalloyl-quinic acid, and kaempferol.

**Figure 2:**
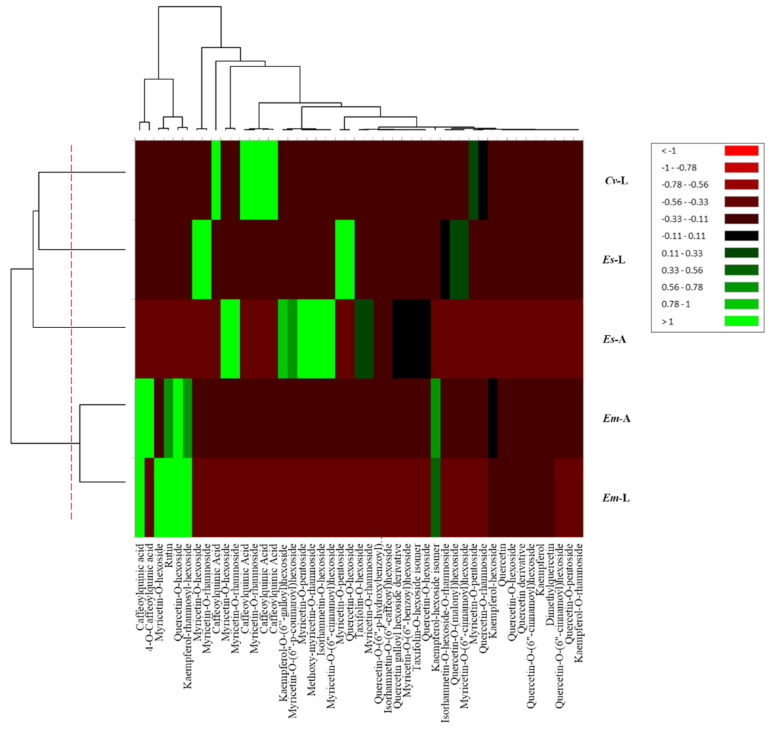
Heat map analysis of phenolic compounds (mean, N = 3) in leaves and aerial parts of 3 different *Ericaceae* taxa: *C. vulgaris* leaves (*Cv*-L), *E. scoparia* leaves (*Es*-L), *E. scoparia* aerial parts (*Es*-A), *E. multiflora* aerial parts (*Em*-A), *E. multiflora* leaves (*Em*-L).

**Figure 3:**
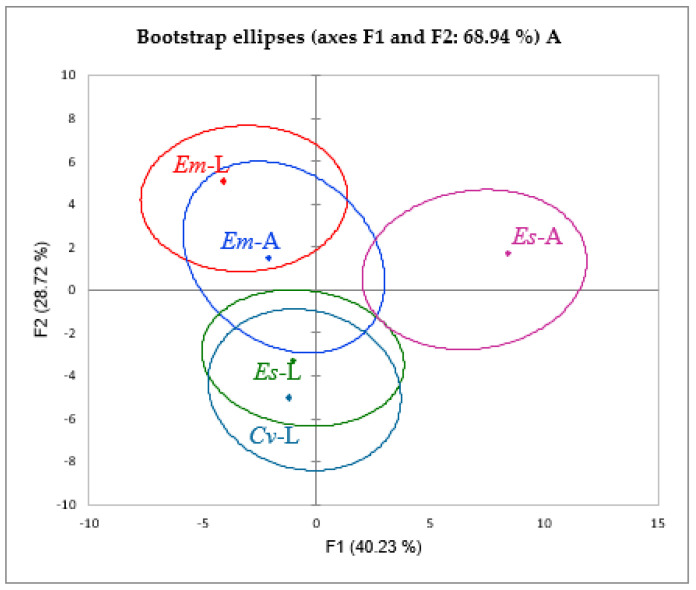

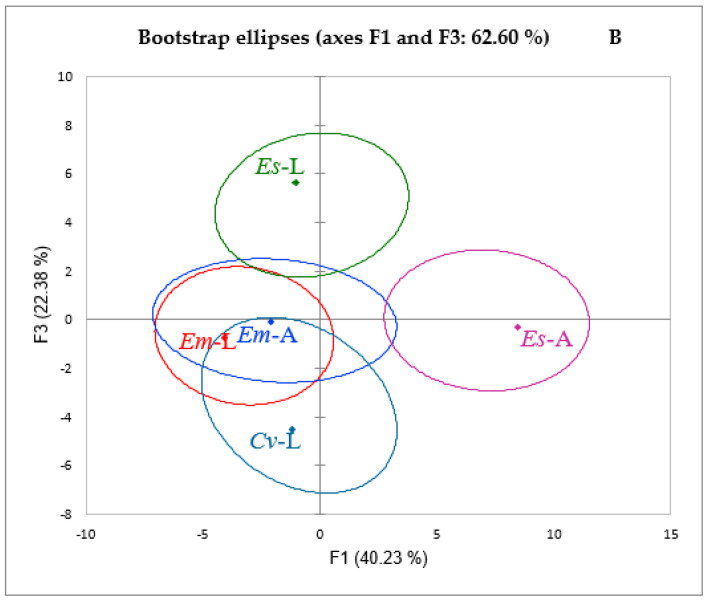
The correlation between phenolic compounds (variables) and plant parts of *Ericaceae* taxa (observations) through PCA. (**A**) represents the first two factorials F1xF2. (**B**) represents the second two factorials F1xF3.

A principal component analysis (PCA) alongside a heat map analysis were carried out on the phenolic compounds as variables to identify the connection between all the plant parts under observation (Figure 2 and Figure 3). The PCA results presented two main components (F1 × F2) that determine 68.94%, whereas (F1 × F3) showed a contribution of 62.60%.

Both statistical analyses confirmed the presence of four different clusters: the first cluster regrouped both parts of *E. multiflora*, and the second and the third clusters were attributed to *E. scoparia* parts, while a completely distinguished fourth cluster was ascribed to *C. vulgaris* leaves. According to the principal components F1 and F2, the leaves of *E. scoparia* and *C. vulgaris* showed a false positive correlation, resulting in a unique cluster, whereas F1 and F3 led to the rejection of the previous correlation and the presence of two different clusters.

### 2.2. Antioxidant and Cytotoxic Activities

#### 2.2.1. Antioxidant Activity

The human body is constantly dealing with the formation of free radicals. When produced in excess, the latter trigger oxidative stress, causing serious tissue injuries. It is well known that many diseases are closely related to oxidative stress, mainly cancer and neurodegenerative disorders (Alzheimer’s, Parkinson’s, etc.). To cope with these health issues, plants provide a cheap and affordable source of natural antioxidants to prevent free radical-induced diseases, especially in countries with low incomes and limited healthcare resources [28]. Many primary antioxidant chemistry reactions can be grouped into the categories of hydrogen-atom transfer (HAT) and single-electron transfer (SET). The HAT mechanism occurs when an antioxidant compound scavenges free radicals by donating hydrogen atoms; the SET mechanism is based on the transfer of a single electron to reduce any compound, including metals, carbonyls, and free radicals [29,30]. It has been reported that, even if many antioxidant reactions are characterized as following either HAT or SET chemical processes, these reaction mechanisms can simultaneously occur [29,31,32].

Due to the complex nature of phytochemicals and their interactions, the importance of using various methods based on different mechanisms for a comprehensive study of the antioxidant properties of plant extracts has been argued. Therefore, the antioxidant activity of *Em*-L, *Em*-A, *Es*-L, *Es*-A, and *Cv*-L extracts was investigated by three different in vitro methods: in order to establish the primary antioxidant properties, the 1,1-diphenyl-1-picrylhydrazyl (DPPH) test, involving HAT and SET mechanisms, and the reducing power, a SET-based assay, were used. The secondary antioxidant properties were determined through the estimation of the ferrous ion (Fe^2+^) chelating activity.

**Figure 4:**
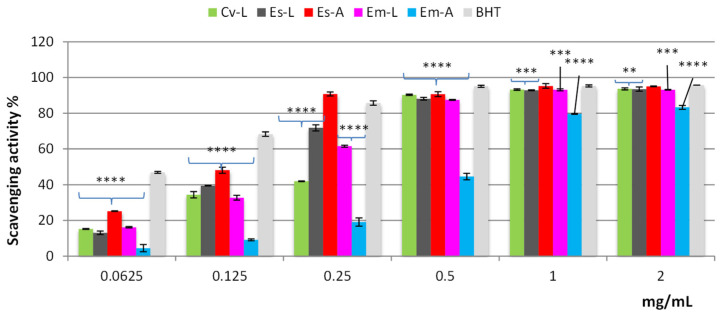
Free radical scavenging activity (DPPH test) of hydroalcoholic extracts from leaves and aerial parts of 3 different *Ericaceae* taxa: *C. vulgaris* leaves (*Cv*-L), *E. scoparia* leaves (*Es*-L), *E. scoparia* aerial parts (*Es*-A), *E. multiflora* leaves (*Em*-L), *E. multiflora* aerial parts (*Em*-A). Data are expressed as the mean ± SD of three independent experiments (*n* = 3) and were analyzed by one-way ANOVA followed by Dunnett’s post-hoc test. **** *p* < 0.0001, *** *p* < 0.001, ** *p* < 0.05 vs. BHT.

The DPPH test is a rapid, simple, inexpensive, and widely used method to measure the free radical scavenging ability of pure compounds or phytocomplexes. Based on the results shown in Figure 4, all extracts, except for *Em*-A, demonstrated valuable radical scavenging activity, reaching approximately 90% of inhibition at the concentration of 0.5 mg/mL. Among the tested extracts, *Es*-A was the most active, as confirmed also by the lowest IC_50_ value (*p* < 0.001); at the concentration of 0.25 mg/mL, it showed activity higher than that of BHT, used as a standard drug, displaying radical scavenging activity superimposable to that of the standard (around 100%) at the concentrations of 1 and 2 mg/mL (Figure 4).

Based on the IC_50_ values, the efficacy of the extracts and the standard decreases in the order *Es*-A > BHT > *Es*-L > *Em*-L > *Cv*-L > *Em*-A (Table 2); however, at 1 mg and 2 mg/mL, *Es*-L, *Em*-L and *Cv*-L exhibited radical scavenging activity close to that of BHT, while only *Em*-A reached about 80% of inhibition (Figure 4).

**Figure 5:**
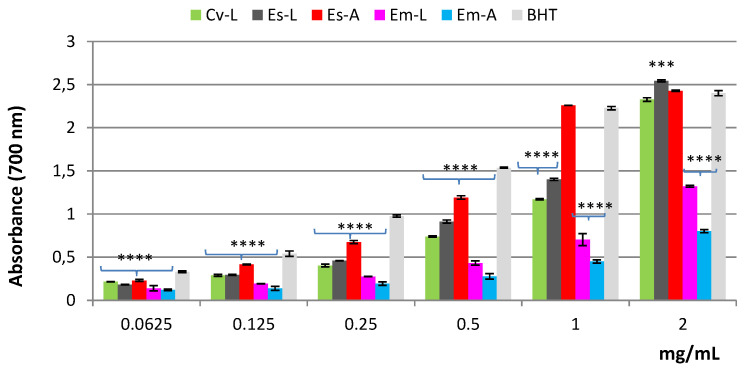
Reducing power of hydroalcoholic extracts from leaves and aerial parts of 3 different *Ericaceae* taxa evaluated by spectrophotometric detection of Fe^3+^-Fe^2+^ transformation method. *C. vulgaris* leaves (*Cv*-L), *E. scoparia* leaves (*Es*-L), *E. scoparia* aerial parts (*Es*-A), *E. multiflora* leaves (*Em*-L), *E. multiflora* aerial parts (*Em*-A). Data are expressed as the mean ± SD of three independent experiments (*n* = 3) and were analyzed by one-way ANOVA followed by Dunnett’s post-hoc test. **** *p* < 0.0001, *** *p* < 0.001, vs. BHT.

The reducing power reflects the ability to stop the radical chain reaction. In this assay, the presence of antioxidant compounds in the sample determines the reduction of Fe^3+^ to the ferrous form (Fe^2+^). As shown in Figure 5, all the extracts, except *Em*-A, displayed good reducing power, which was dose-dependent. Among the tested extracts, those of *E. scoparia* were the most active. In fact, at the concentration of 1 mg/mL, *Es*-A showed activity close to that of BHT; at 2 mg/mL, the reducing power of both *Es*-A and *Es*-L was higher than that of the standard. Based on the ASE/mL values, the efficacy of the extracts and the standard decreases in the order BHT > *Es*-A > *Es*-L > *Cv*-L > *Em*-L > *Em*-A (Table 2).

The Fe^2+^ chelating activity of *Em*-L, *Em*-A, *Es*-L, *Es*-A, and *Cv*-L extracts was estimated by monitoring the formation of the Fe^2+^-ferrozine complex. In this assay, only *Es*-A and *Em*-A displayed weak chelating properties, whereas all the other extracts were not active (Table 2).

From our findings, it is evident that all the extracts possess strong primary antioxidant properties; interestingly, that obtained from the aerial parts of *E. scoparia* is the most powerful. HPLC analysis revealed, for this extract, the highest content of flavonoid compounds, represented mainly by flavonols such as several myricetin glycosides, but also kaempferol, quercetin, and isorhamnetin glycosides. The flavonols, containing more hydroxyl groups (one to six OH groups), have a very strong ability to scavenge DPPH radicals and they are well-known, potent antioxidants. These compounds have a 3-hydroxyl group in the C-ring and *3′,4′*-dihydroxy groups (catechol structure) in the B-ring, but also possess the 2,3-double bond in conjugation with the 4-oxo function in the C-ring, which are the essential structural elements for potent radical scavenging activity [33].

*Erica scoparia* aerial part extract is rich in myricetin glycosides, which have been shown to possess strong primary antioxidant activity [34,35]. Thus, the best activity observed for *Es*-A could be correlated primarily to these compounds, but also to kaempferol, isorhamnetin, and quercetin glycosides.

#### 2.2.2. *Artemia salina* Lethality Bioassay

The toxicity of *Em*-L, *Em*-A, *Es*-L, *Es*-A, and *Cv*-L extracts was assessed by the *Artemia salina* lethality bioassay, extensively utilized as an alternative model for toxicity evaluation. This simple method offers numerous advantages, such as rapidity, low cost, continuous availability of cysts (eggs), and ease of maintenance under laboratory conditions [36]. It is a useful system for predicting the toxicity of plant extracts in order to consider their safety. The results of the bioassay showed that the median lethal concentration values were higher than 1000 µg/mL for all the tested extracts, thus indicating the lack of toxicity against brine shrimp larvae based on Clarkson’s toxicity criterion [37].

## 3. Materials and Methods

### 3.1. Chemicals and Reagents

LC–MS-grade water (H_2_O), acetonitrile (ACN), formic acid, methanol, and DMSO were purchased from Merck Life Science (Merck KGaA, Darmstadt, Germany). Taxifolin, rutin, 4-caffeoylquinic acid, ishorhamnetin, quercetin, and kaempferol-3-glucoside were also obtained from Merck Life Science (Merck KGaA, Darmstadt, Germany). Unless indicated otherwise, all chemicals were purchased from Sigma-Aldrich (Milan, Italy).

### 3.2. Plant Materials

Three *Ericaceae* taxa, *Erica multiflora*, *Erica scoparia*, *Calluna vulgaris*, were collected in December 2019 from three different places in Northern Morocco; Khemiss anjra (Tetouan province) with longitude −5.5125257, latitude 35.6632287; Ben karrich (Tetouan province), longitude −5.4279948, latitude 35.5068513; Souq l’qolla (Chefchouaen), longitude −5.59873, latitude 35.12112 35, respectively. The taxonomic identification was confirmed by Prof. Kadiri Mohamed, Abdelmalek Essaadi University, Tetouan, Morocco.

The plant material was collected in December according to their flourishing stage. The selected samples for the preparation of the extracts consisted of leaves and aerial parts, for both *Erica* species that bloomed in this month, while, for *C. vulgaris*, only the leaves were used because, in the same period, this species had not yet flowered.

The selected parts were dried in darkness at room temperature for 2 weeks, and then crushed in an electrical grinder to a particle size less than 4 mm; the grounded parts were stored in a refrigerator under 4 °C in amber glass vials to avoid oxidation effects.

### 3.3. Extraction Procedure

One hundred milligrams of different powdered plant material of the three studied species was extracted, in a 50 mL volumetric flask, with 10 mL of ethanol:water, 96:4 (*v:v*), followed by sonication (60 W, 25 °C, 37 Hz) for 20 min. The obtained extracts were centrifugated for 10 min under 3000 rpm and filtered using Whatman filter paper (Merck Life Science, Merck KGaA, Darmstadt, Germany). The extraction procedure was repeated three times, and then the filtrates were combined, evaporated to dryness by a rotavapor and stored at 4 °C. The yields of the extracts, referring to 100 g of dried plant material, were 31.37% for *E. multiflora* leaves (*Em*-L), 33.26% for *E. multiflora* aerial parts (*Em*-A), 37.97% for *E. scoparia* leaves (*Es*-L), 46.76% for *E. scoparia* aerial parts (*Es*-A), and 33.72% for *C. vulgaris* leaves (*Cv*-L).

### 3.4. LC–DAD/ESI–MS Analyses

The hydroalcoholic extracts (*Em*-L, *Em*-A, *Es*-L, *Es*-A, and *Cv*-L) were analyzed through the LC–MS technique using a Shimadzu liquid chromatography system (Kyoto, Japan), composed of a CBM-20A controller, two LC-30AD dual-plunger parallel-flow pumps, a DGU-20A5R degasser, a CTO-40C column oven, a SIL-40C autosampler, an SPD-M40 photo diode array detector, and an LCMS-8050 mass spectrometer, through an ESI source (Shimadzu, Kyoto, Japan).

Separation analyses were performed on a 150 × 4.6 mm; 2.7 µm Ascentis Express RP C18 column (Merck Life Science, Merck KGaA, Darmstadt, Germany). The mobile phase was composed of two solvents, water (solvent A) and acetonitrile (solvent B), both acidified with formic acid at 0.1% *v*/*v*. The flow rate was set at 1 mL/min and a simplified linear gradient of elution program was followed: 0–5 min, 0–30% B, 5–30 min, 30–100% B, 35 min, 100% B. PDA range: 200–400; λ = 280 nm (sampling frequency: 40.0 Hz, time constant: 0.08 s).

The applied mass spectrometry conditions were as follows: scan range, *m/z* 100–1200; scan speed, 2500 amu/s; event time, 0.3 s; nebulizing gas (N_2_) flow rate, 1.5 L/min; drying gas (N_2_) flow rate, 15 L/min; interface temperature, 350 °C; heat block temperature, 300 °C; DL (desolvation line) temperature, 300 °C; DL voltage, 1 V; interface voltage, −4.5 kV.

### 3.5. Preparation of Calibration Curves

Calibration curves of six polyphenolic standards (R² > 0.9989) were used for the quantification of the polyphenolic content in sample extracts by using different concentration levels: 4-caffeoylquinic acid (y = 3450.1x − 26,363; LoD = 0.034, LoQ = 0.104), taxifolin (y = 18,001x − 35,329; LoD = 0.071, LoQ = 0.215), rutin (y = 10,066x + 2176.5; LoD = 0.014, LoQ = 0.042), isorhamnetin (y = 25,334x + 1890.3; LoD = 0.116, LoQ = 0.353), quercetin (y = 20,376x + 7053.8, LoD = 0.007, LoQ = 0.022), kaempferol-3-glucoside (y = 13,848x + 2354.1, LoD = 0.090, LoQ = 0.274). Each analysis was performed in triplicate.

### 3.6. Antioxidant and Cytotoxic Activities

#### 3.6.1. Free Radical Scavenging Activity

The free radical scavenging activity of *Em*-L, *Em*-A, *Es*-L, *Es*-A, and *Cv*-L extracts was determined using the DPPH (1,1-diphenyl-1-picrylhydrazyl) method [38]. The samples were tested at different concentrations (0.0625–2 mg/mL). An aliquot (0.5 mL) of solution containing different amounts of sample was added to 3 mL of daily prepared methanol DPPH solution (0.1 mM). The optical density change at 517 nm was measured, 20 min after the initial mixing, with a model UV-1601 spectrophotometer (Shimadzu). Butylated hydroxytoluene (BHT) was used as reference.

The scavenging activity was measured as the decrease in the absorbance of the samples versus DPPH standard solution. Results were expressed as the radical scavenging activity percentage (%) of the DPPH, defined by the formula [(Ao − Ac)/Ao] × 100, where Ao is the absorbance of the control and Ac is the absorbance in the presence of the sample or standard.

The results, obtained from the average of three independent experiments, are reported as mean radical scavenging activity percentage (%) ± standard deviation (SD) and mean 50% inhibitory concentration (IC_50_) ± SD. The IC_50_ value is a parameter calculated as the concentration of extract needed to decrease the initial DPPH concentration by 50%. Thus, the lower IC_50_ value, the higher the antioxidant activity of the sample.

#### 3.6.2. Reducing Power Assay

The reducing power of *Em*-L, *Em*-A, *Es*-L, *Es*-A, and *Cv*-L extracts was evaluated by the spectrophotometric detection of Fe^3+^-Fe^2+^ transformation method [39]. The extracts were tested at different concentrations ranging from 0.0625 to 2 mg/mL. Solutions of different concentrations of extracts in 1 mL solvent were mixed with 2.5 mL of phosphate buffer (0.2 M, pH 6.6) and 2.5 mL of 1% potassium ferricyanide [K_3_Fe(CN)_6_], and the resulting mixture was incubated at 50 °C for 20 min. The solution was cooled rapidly, mixed with 2.5 mL of 10% trichloroacetic acid, and centrifuged at 3000 rpm for 10 min. After centrifugation, the supernatant (2.5 mL) was mixed with 2.5 mL of distilled water and 0.5 mL of 0.1% fresh ferric chloride (FeCl_3_). The absorbance of the solution was measured at a wavelength of 700 nm after 10 min. An increase in the absorbance of the reaction mixture indicates an increase in its reducing power. An equal volume (1 mL) of water mixed with a solution prepared as described above was used as a blank. Ascorbic acid and BHT were used as references. The results averaged from three independent experiments were expressed as mean absorbance values ± SD. The reducing power was also expressed as ascorbic acid equivalent (ASE/mL); when the reducing power is 1 ASE/mL, the reducing power of 1 mL extract is equivalent to 1 μmol ascorbic acid.

#### 3.6.3. Ferrous Ion (Fe^2+^) Chelating Activity

The Fe^2+^ chelating activity of *Em*-L, *Em*-A, *Es*-L, *Es*-A, and *Cv*-L extracts was estimated according to the method reported by Decker and Welch [40]. The samples were tested at different concentrations (0.0625–2 mg/mL). Briefly, different concentrations of each sample in 1 mL solvent were mixed with 0.5 mL of methanol and 0.05 mL of 2 mM FeCl_2_. The reaction was initiated by the addition of 0.1 mL of 5 mM ferrozine. Then, the mixture was shaken vigorously and left standing at room temperature for 10 min. The absorbance of the solution was measured spectrophotometrically at 562 nm. The control contained FeCl_2_ and ferrozine, complex formation molecules. Ethylenediaminetetraacetic acid (EDTA) was used as a reference. The percentage of inhibition of the ferrozine—(Fe^2+^) complex formation was calculated by the formula [(Ao − Ac)/Ao] × 100, where Ao is the absorbance of the control and Ac is the absorbance in the presence of the sample or standard. The results, obtained from the average of three independent experiments, are reported as mean inhibition of the ferrozine—(Fe^2+^) complex formation (%) ± SD and IC_50_ ± SD.

#### 3.6.4. *Artemia salina* Lethality Bioassay

The potential toxicity of *Em*-L, *Em*-A, *Es*-L, *Es*-A, and *Cv*-L L extracts was investigated in brine shrimp (*Artemia salina* Leach) [41]. Ten brine shrimp larvae, taken 48 h after initiation of hatching in artificial seawater, were transferred to each sample vial, and then artificial seawater was added to obtain a final volume of 5 mL. Different concentrations of each extract were added (10–1000 µg/mL) and the brine shrimp larvae were incubated for 24 h at 25–28 °C. Then, the surviving larvae were counted using a magnifying glass. The assay was carried out in triplicate, and median lethal concentration (LC_50_) values were determined by Litchfield and Wilcoxon’s method. Extracts giving LC_50_ values greater than 1000 µg/mL were considered non-toxic.

### 3.7. Statistical Analysis

The heat map and PCA were established to provide an easier comparison of the phenolic compounds between the plant parts; the results were expressed as mean values ± relative standard deviation (RSD). All data were processed with principal component analysis (PCA) and collected in a heat map; the phenolic compounds were considered as variables in these plots to identify the connections between all the plant parts as observations. Principal component analysis (PCA) and heat map were generated using XLSTAT software ver. 2019.2.2.

Statistical comparison of the antioxidant activity data was carried out by using one-way analysis of variance (ANOVA) (GraphPAD Prism Version 9.4.0. Software for Science). *p*-values lower than 0.05 were considered statistically significant.

## 4. Conclusions

In this contribution, three Moroccan *Ericaceae* species, namely *Erica multiflora, Erica scoparia*, and *Calluna vulgaris*, were investigated. The phenolic profiles of the leaf and aerial extracts revealed a quite complex pattern, with up to 52 phenolic compounds positively identified, including phenolic acids and flavonoids. The antioxidant properties of the extracts were evaluated by means of three different methods, namely DPPH, reducing power, and Fe^2+^ chelating assays, demonstrating their high potential. On the basis of the phenolic profile and remarkable results achieved for the antioxidant activity, such species could be considered as a potential safe source of bioactive compounds to be advantageously employed in traditional Moroccan medicine. Interestingly, myricetin derivates might have important therapeutic potential, e.g., antioxidant, anti-inflammatory, anti-diabetes, anticancer, and protective effects against Alzheimer’s disease [42]; furthermore, the efficacy kaempferol and rutin can be exploited against doxorubicin-induced cardiotoxicity [43], while quercetin could be employed for its interesting anticancer effects against prostate and breast cancers [44].

## Figures and Tables

**Table 1:** Phenolic compounds detected in *C. vulgaris*, *E. multiflora*, and *E. scoparia*.

Peak No	Compound	t_R_ (min)	UV max (nm)	[M-H]-	*E. multiflora* (mg/Kg ± RSD%)		*E. scoparia* (mg/Kg ± RSD%)		*C. vulgaris* (mg/Kg ± RSD%)
					Leaves	Aerial Parts	Leaves	Aerial Parts	Leaves
1	Taxifolin-*O*-hexoside	4.11	288	465, 303, 313				332.96 ± 0.68	
2	Taxifolin-*O*-hexoside isomer	4.24	284	465, 303, 313				214.93 ± 1.49	
3	Digalloyl-quinic acid	4.61	274	495				Nq	
4	Caffeoylquinic acid	4.81	297sh, 326	353, 191, 179	53.93 ± 0.11	61.11 ± 0.18			
5	4-*O*-Caffeoylquinic acid	4.91	297sh, 326	353, 191, 179		83.75 ± 0.74			
6	Caffeoylquinic acid	4.99	290, 325	353, 191,137					626.40 ± 0.77
7	Myricetin-*O*-hexoside	5.38	258, 358	479, 317				2130.25 ± 0.78	
8	Eriodictyol-*O*-hexoside	5.42	297, 321	449, 287		Nq			
9	Caffeoylquinic acid	5.42	290, 325	353, 191,137					138.37 ± 0.23
10	Caffeoylquinic acid	5.47	290, 325	353, 191,137					231.54 ± 1.68
11	Quercetin derivative	5.63	260, 356	615, 463, 301	2.89 ± 0.83				
12	Myricetin-*O*-hexoside isomer	5.67	356	479, 317	43.46 ± 0.35				
13	Myricetin-*O*-pentoside	5.70	259, 357	449, 317				852.85 ± 1.97	
14	Myricetin-*O*-rhamnoside	5.74	260, 357	463, 317				1625.89 ± 0.39	
15	Quercetin-*O*-hexoside	5.83	255, 353	463, 301				213.14 ± 0.43	
16	Rutin	5.87	257, 354	609, 301	55.44 ± 2.59	14.16 ± 0.18			
17	Caffeoylquinic acid	5.87	290, 325	353, 191,137					184.69 ± 0.95
18	Methoxy-myricetin-*O*-rhamnoside	5.88	254, 358	493				810.78 ± 0.43	
19	p-Coumaroylquinic acid	6.07	312	337					Nq
20	Quercetin-*O*-hexoside	6.08	255, 355	463, 301	117.43 ± 0.48	29.48 ± 1.76			
21	Quercetin-*O*-hexoside	6.13	354	463, 301	4.78 ± 0.67	0.10 ± 2.51			
22	Kaempferol-*O*-(6″-galloyl)hexoside	6.17	253, 358	599, 285				564.64 ± 0.19	
23	Myricetin-*O*-rhamnoside	6.20	358	463				268.52 ± 0.08	
24	Myricetin-*O*-hexoside	6.21	356	479, 317			184.38 ± 0.26		
25	Kaempferol-rhamnosyl-hexoside	6.24	264, 347	593, 447, 285	90.76 ± 1.19	15.24 ± 0.21			
26	Myricetin-*O*-hexoside	6.25	356	479, 317					41.66 ± 1.88
27	Isorhamnetin-*O*-hexoside	6.32	252, 357	477				683.43 ± 0.93	
28	Kaempferol-hexoside	6.51	264, 348	447, 285	4.83 ± 1.27	5.55 ± 2.06			
29	Myricetin-*O*-pentoside	6.55	260, 357	449, 317			72.79 ± 0.05		
30	Quercetin galloyl hexoside derivative	6.56	357	615				160.67 ± 1.25	
31	Myricetin-*O*-pentoside	6.59	281, 349	449, 317					48.81 ± 2.22
32	Kaempferol-hexoside isomer	6.61	264, 348	447, 285	17.08 ± 0.35	14.53 ± 0.44			
33	Myricetin-*O*-rhamnoside	6.65	260, 357	463, 317			153.65 ± 1.13		
34	Quercetin-*O*-hexoside	6.68	255, 353	463, 301			64.25 ± 1.47		2.82 ± 3.24
35	Myricetin-*O*-(6″-benzoyl)hexoside	6.70	265,316, 358	583, 316				200.83 ± 0.20	
36	Methyl-ellagic acid hexoside	6.72	283	477		Nq			
37	Myricetin-*O*-rhamnoside	6.72	260, 357	463, 317					232.98 ± 0.35
38	Unknown	6.97	344	649		Nq			
39	Quercetin-*O*-(malonyl)hexoside	7.03	356	549			18.52 ± 0.27		
40	Quercetin-*O*-pentoside	7.06	255, 354	433, 301					9.44 ± 0.28
41	Unknown	7.11	358	599, 507, 463			Nq		
42	Quercetin-*O*-(6″-p-hydroxybenzoyl) hexoside	7.17	269, 356	583, 316				91.34 ± 1.22	
43	Unknown	7.22	350	723, 677, 477			Nq		
44	Quercetin-*O*-rhamnoside	7.22	255, 342	447, 301					32.30 ± 0.02
45	Kaempferol-*O*-rhamnoside	7.77	263, 341	431, 285					18.77 ± 0.55
46	Unknown	7.89	312	731		Nq			
47	Myricetin-*O*-(6″-cinnamoyl)hexoside	8.14	265, 359	609, 317, 301				757.33 ± 1.96	
48	Unknown	8.22	288, 308	289					Nq
49	Unknown	8.30	309	483, 289					Nq
50	Quercetin-*O*-(6″-cinnamoyl)hexoside	8.41	281	593, 447, 301	3.72 ± 1.10				
51	Quercetin	8.66	268, 370	301	3.34 ± 2.11	1.39 ± 5.97			
52	Myricetin-*O*-(6″-p-coumaroyl)hexoside	8.77	265, 360	624				509.39 ± 0.94	
53	Isorhamnetin-*O*-(6″-caffeoyl)hexoside	9.45	264, 359	639				111.98 ± 0.50	
54	Dimethylquercetin	9.46	227, 344	329, 301	0.86 ± 8.95	1.38 ± 0.62			
55	Myricetin-*O*-(6″-cinnamoyl)hexoside	9.53	264, 359	609, 317, 301			23.46 ± 1.49		
56	Kaempferol	10.22	366	285	0.49 ± 1.89	0.91 ± 1.84			
57	Isorhamnetin-*O*-hexoside-*O*-rhamnoside	10.22	264, 359	623			9.76 ± 0.34		
58	Quercetin-*O*-(6″-cinnamoyl)hexoside	10.40	356	593, 447, 301			0.79 ± 1.12		
59	Unknown	10.97	356	637, 347			Nq		
	Total				399.01 ± 1.46	227.6 ± 0.15	527.6 ± 1.55	9528.93 ± 54.32	1567.78 ± 13.01

Nq: Not quantified.

**Table 2:** Free radical scavenging activity (DPPH test), reducing power, and ferrous ion (Fe^2+^) chelating activity of hydroalcoholic extracts from leaves and aerial parts of 3 different *Ericaceae* taxa.

*Ericaceae* Taxa	DPPH Test IC_50_ (mg/mL)	Reducing Power ASE/mL	Fe^2+^ Chelating Activity IC_50_ (mg/mL)
*Cv*-L	0.212 ± 0.061 ^a^	2.790 ± 0.100 ^a^	NA
*Es*-L	0.189 ± 0.051 ^a^	2.721 ± 0.062 ^a^	NA
*Es*-A	0.142 ± 0.014 ^b^	1.898 ± 0.056 ^b^	>2
*Em*-L	0.200 ± 0.001 ^a^	3.814 ± 0.091 ^c^	NA
*Em*-A	0.611 ± 0.017 ^c^	5.538 ± 0.148 ^d^	>2
Standard	BHT 0.154 ± 0.001 ^b^	BHT 1.131 ± 0.037 ^e^	EDTA 0.0067 ± 0.0003

*C. vulgaris* leaves (*Cv*-L), *E. scoparia* leaves (*Es*-L), *E. scoparia* aerial parts (*Es*-A), *E. multiflora* leaves (*Em*-L), *E. multiflora* aerial parts (*Em*-A). NA: no activity. Data are expressed as the mean ± SD of three independent experiments (*n* = 3) and were analyzed by one-way ANOVA followed by Tukey–Kramer multiple comparisons test. ^a–e^ Different letters within the same column indicate significant differences between mean values (*p* < 0.001).

## Data Availability

Not applicable.
